# The effects of 8-week complex training on lower-limb strength and power of Chinese elite female modern pentathlon athletes

**DOI:** 10.3389/fpsyg.2022.977882

**Published:** 2022-10-25

**Authors:** Zining Qiao, Zhenxiang Guo, Bin Li, Meng Liu, Guozhen Miao, Limingfei Zhou, Dapeng Bao, Junhong Zhou

**Affiliations:** ^1^School of Strength and Conditioning Training, Beijing Sport University, Beijing, China; ^2^Nanjing University of Aeronautics and Astronautics, Nanjing, China; ^3^Cycling and Fencing Sports Administrative Center Under the General Administration of Sport of China, Beijing, China; ^4^Sports Coaching College, Beijing Sport University, Beijing, China; ^5^Maranatha High School, Pasadena, CA, United States; ^6^China Institute of Sport and Health Science, Beijing Sport University, Beijing, China; ^7^Harvard Medical School, Hebrew SeniorLife Hinda and Arthur Marcus Institute for Aging Research, Boston, MA, United States

**Keywords:** complex training, lower extremity, power, modern pentathlon, female athletes

## Abstract

Complex training (CT) is a combination training method that alternates between performing high-load resistance training (RT) and plyometric training within one single session. The study aimed to examine the effects of CT on lower-limb strength and power of elite female modern pentathlon athletes under the new modern pentathlon format and competition rules. Ten female participants (age: 23.55 ± 2.22 years, weight: 60.59 ± 3.87 kg, height: 169.44 ± 4.57 cm, and training experience: 6.90 ± 2.08 years) of the national modern pentathlon team completed 8 weeks of RT as followed by 8 weeks of CT, with 2 weeks of break. Then, the participants conducted 8 weeks of CT, which included RT combined with plyometric training (e.g., drop jump and continuous jump). All stages of training were designed by the linear strength training period theories, requiring participants to train twice for the first 4 weeks and three times for the second 4 weeks. The one-repetition maximum (1RM) of squat, isometric mid-thigh pull (IMTP), counter-movement jump (CMJ), squat jump (SJ), pre-stretch augmentation percentage (PSAP), and reaction strength index (RSI) were assessed before and after both RT and CT training. One-way repeated-measure ANOVA models revealed that the 1RM of squat was significantly improved (*p* < 0.001) after RT as compared to pre-RT. No significant improvement in IMTP (*p* = 0.055), CMJ (*p* = 0.194), SJ (*p* = 0.692), PSAP (*p* = 0.087), and RSI (*p* = 0.238) was not observed. After CT, 1RM of squat (*p* < 0.001), IMTP (*p* < 0.035), CMJ (*p* < 0.001), SJ (*p* < 0.008), RSI (*p* < 0.006) were significant improved as compared to pre-RT, post-RT and pre-CT, while significant improvements in PSAP were observed as compared to pre-RT (*p* = 0.003) and pre-CT (*p* = 0.027), but not to post-RT (*p* = 0.156). This pilot study showed the promise of CT following RT to improve lower-limb strength and power in elite female modern pentathlon athletes. The findings are worthwhile to be confirmed in future studies with larger sample size and randomized design.

## Introduction

The modern pentathlon consists of fencing, swimming, equestrian, and a combined event of shooting and cross country running (i.e., laser-run), requiring skilled technique and multiple physical fitness (Le Meur et al., 2010). Lower-limb strength and power are one of the most important contributors to the performance of modern pentathlon (Chirico et al., 2019). Ko et al. (2021) observed that, for example, lower-limb strength and power (e.g., the average strength of flexor muscle and extensor muscle, squat, and sergeant jump) is the most relevant predictor of overall modern pentathlon performance, especially in the fencing, swimming, and laser-run.

Additionally, the International Union of Modern Pentathlon (UIPM) recently has published the new modern pentathlon format and competition rules for 2022–2024, which shortened the distance of laser-run event (from 4 × 800 m to 5 × 600 m), and adopted the short course of swimming (from 50 m to 25 m; Union Internationale de Pentathlon Moderne, 2022). Compared to old rules, these newly-established rules require athletes to have greater lower-limb strength and power to complete the short-distance competition (Hanon and Thomas, 2011; Berryman et al., 2018). These rule changes further highlight the importance of lower-limb strength and power for the competition performance in modern pentathlon, which may thus serve as the appropriate target for strategies aiming to improve combined performance of modern pentathlon athletes.

One such strategy is resistance training (RT), including basic strength training (e.g., squat, split squat, deadlift, etc.). Previous studies found that RT can improve strength performance (e.g., one-repetition maximum [1RM] of squat) in athletes of different sports, such as swimming (Crowley et al., 2017), 800-m running (Bachero-Mena et al., 2021), and fencing (Turner et al., 2014). However, a recent study had shown that RT enhanced muscle hypertrophy but not strength in trained men due to a certain threshold of RT, over which further training in RT was not advantageous and might only delay recovery from exercises for resistance trained-individual (e.g., elite athletes; Schoenfeld et al., 2019). Thereby, these may necessitate RT protocol to combine other training methods to augment its benefits. Complex training (CT) is a combination training method that alternates between performing high-load RT and plyometric training within one single session. The CT can elicit a post-activation potentiation (PAP) response to induce more power in the subsequent plyometric training for athletes (Carter and Greenwood, 2014; Clemente et al., 2021), by stimulating high-order motor unit recruitment and excitability, increasing phosphorylation of the myosin light chain, and changes in limb stiffness (Tillin and Bishop, 2009; Blagrove et al., 2019). Using CT can induce significant improvement of neuromuscular adaptation (MacDonald et al., 2012), and strength and power of muscle (Berriel et al., 2022; Liu et al., 2022). In addition, this CT can simultaneously enhance multiple aspects contributing to running performance, such as running economy and maximal running speed (Blagrove et al., 2019; Li et al., 2019). Taken together, CT may be of great promise to benefit the athletic performance of female modern pentathlon athletes by simultaneously augmenting their lower-limb strength and power, which, however, have not been examined.

This pilot study thus aims to examine if an 8-week CT following RT can augment the lower-limb strength and power in a group of elite female modern pentathlon athletes. Specifically, we hypothesize that the CT protocol would induce a significant increase in athletic performance pertaining to lower-limb strength and power that cannot be enhanced using RT.

## Materials and methods

### Participants

Ten elite female modern pentathlon participants volunteered to participate in this study (Table 1). The inclusion criteria were as follows: (1) Participants who were preparing for the 19th Asian Games Hangzhou 2022; (2) participants who were proficient in resistance and plyometric training techniques; and (3) the ability and willingness to complete 16-week training programs of tests and intervention. The exclusion criteria were as follows: (1) Participants suffered from severe lower-body injuries related to anterior cruciate ligament, hamstring, meniscus, and ankle, or any medical and orthopedic problems during the last 3 years and (2) unable to execute plyometric exercises. Before data collection, the participants were informed about the benefits and possible risks associated with the study, and the participants provided written informed consent to participate. The participants were on the same routine of diet without taking additional nutritional supplement and had caffeine-free beverages during the whole study period. The study protocol was approved by the Beijing Sport University Institutional Research Commission (approval number: 2022132H), and all procedures were conducted in accordance with the Declaration of Helsinki.

**Table 1:** The demographic information of the participants.

	Age (years)	Height (cm)	Weight (kg)	Training experience (years)	BMI	Body fat (%)
Participants (*N* = 10)	23.55 ± 2.22	169.44 ± 4.57	60.59 ± 3.87	6.90 ± 2.08	20.80 ± 0.99	23.16 ± 2.32

### Procedures

The participants first completed the RT for 8 weeks and then the CT for 8 weeks. Table 2 shows the details description and progression of RT and CT programs. Between RT and CT, a 2-week resting period was given. According to the liner strength training periods principles, three phases in each training program were completed: phase 1 of 1 week aimed to improve the anatomical adaptations of muscles, phase 2 of 3 weeks aimed to enhance the hypertrophy of muscles, and phase 3 of 4 weeks was to improve maximal strength and power. Participants were required to train twice in phase 1 and phase 2, and three times for phase 3 with a 48 ~ 72 h of recovery between each training session. Traditional mainly focuses on structural exercises of large muscle groups (e.g., squat, deadlift, and hip thrust). The load arrangement of single strength training session adopts linear periodization load (e.g., pyramid training method) to develop nerve function and strength of lower extremities.

**Table 2:** Resistance training and plyometric training program.

Phase	Period	RT (week 1–8)	CT (week 10–18)
Phase 1	1 week	65%1RM × 15RM × 6–8 groups × 60 s	(65%1RM × 15RM^S^ + 3-5RM^P^) × 6–8 groups × 60 s
Phase 2	3 weeks	70–85%1RM × 6-12RM × 6 ~ 8 groups × 90 s	(70–85%1RM × 6 ~ 12RM^S^ + 5-10RM^P^) × 6–8 group × 90 s
Phase 3	4 weeks	80–100%1RM × 1-8RM × 6–8 groups × 180–240 s	(80–100%1RM × 1-8RM^S^ + 5-10RM^P^) × 6–8 groups × 18–240 s

RM, maximum repetitions; % 1RM, percentage of 1RM maximum load intensity; S, regular strength training; P, plyometric exercises.

**Table 3:** The assessment results for week 1–8 (RT) and week 10–18 (CT).

	Week 1–8 (RT) mean difference with 95% of confidence intervals				Week 10–18 (CT) mean difference with 95% of confidence intervals			
Variable	Pre-RT	Post-RT	Mean Differences	Cohen’s d	Pre-CT	Post-CT	Mean Differences	Cohen’s d
1RM Squat (kg)	70.40 ± 5.34 (67.1, 73.7)	81.10 ± 3.93 (78.7, 83.5)	10.70 ± 4.63	2.28	76.30 ± 4.22 (73.7, 78.9)	92.50 ± 4.77 (89.5, 95.5)^*^	16.2 ± 2.90	3.60
IMTP (kg)	189.12 ± 15.88 (179, 199)	202.67 ± 13.24 (194, 211)	13.56 ± 8.65	0.93	191.90 ± 14.33 (183, 201)	217.61 ± 17.38 (207, 228)^*^	25.71 ± 7.58	1.61
CMJ (cm)	28.62 ± 3.02 (26.7, 30.5)	30.22 ± 2.71 (28.5, 31.9)	1.60 ± 0.69	0.56	30.10 ± 2.99 (28.2, 32.0)	35.70 ± 1.95 (34.5, 36.9)^*^	5.60 ± 1.80	2.22
SJ (cm)	26.60 ± 3.30 (24.6, 28.6)	27.11 ± 2.79 (25.4,28.8)	0.51 ± 1.23	0.17	27.47 ± 3.08 (25.6, 29.4)	31.06 ± 2.13 (29.7, 32.4)^*^	3.59 ± 2.63	1.36
PSAP (%)	7.16 ± 4.11 (4.61, 9.71)	10.33 ± 3.44 (6.77, 10.80)	3.17 ± 4.62	0.84	8.79 ± 3.25 (8.19, 12.50)	12.93 ± 5.05 (9.80, 16.10)	4.14 ± 6.28	0.97
RSI	1.10 ± 0.24 (0.95, 1.25)	1.23 ± 0.20 (1.10, 1.35)	0.13 ± 0.06	0.59	1.21 ± 0.30 (1.03, 1.40)	1.54 ± 0.20 (1.42, 1.66)^*^	0.32 ± 0.20	1.29

* Statistically significant difference as compared to all the other pre- and post-interventions, *p* < 0.05.

1RM, one repetition of maximum; IMTP, isometric mid-thigh pull; CMJ, counter-movement jump; SJ, squat jump; PSAP, pre-stretch augmentation percentage; RSI, reaction strength index.

During each session, participants received consistent instructions from certified strength and conditioning coaches on proper techniques for resistance and plyometric exercises. All the protocols were designed and supervised by study personnel, who is an experienced researcher in strength and conditioning, and a fitness trainer.

### Test program

Before and immediately after RT (within 3 days after week 8) and CT (within 3 days after week 18), the 1RM of squat, isometric mid-thigh pull (IMTP), counter-movement jump (CMJ), squat jump (SJ), pre-stretch augmentation percentage (PSAP), and reaction strength index (RSI) were assessed. All testing sessions were preceded by a standardized warm-up with included stretching and dynamic movements. There was an interval of 5 min between each test. Before each testing session, participants finished standardized warm-up for 15 min, including 5 min dynamic stretch, 8 min movement integration, and 2 min neural activation.

#### 1RM of squat test

Lower-limb strength was assessed with a 1RM squat as reported by previous studies (Keiner et al., 2013; Jurado-Castro et al., 2022). The maximal load of the parallel back-squat exercise (1RM) was determined using procedures outlined by National Strength and Conditioning Association (NSCA; Miller, 2012). The movement for the parallel back-squat exercise was performed as described above for the squat training. As a squat-exercise warm-up before 1RM measurement, the participates performed 4 sets of squat exercises with (a) 20 kg for 10 repetitions, (b) 50% estimated 1RM for 5 repetitions, (c) 75% estimated 1RM for 3 repetitions, and (d) 90% estimated 1RM for 1 repetition to ensure maximal effort. For determination of workload, the estimated 1RM was used from the participants’ recruitment sessions before participation in this study. Then, they tried the estimated 100% 1RM, and the load was increased by 5–10 kg until failure. The 1RM was determined by 6 attempts. They were provided with 3 min of rest between sets, which was considered to be sufficient.

#### IMTP test

The IMTP test was used to assess the isometric strength of lower limb (Farney et al., 2020). The mid-thigh position was determined for each participant before testing by marking the midpoint distance between the knee and hip joints. Each participant was instructed to assume their preferred deadlift position by self-selecting their hip and knee angles. The height of the barbell was adjusted up or down to make sure it was in contact with the mid-thigh. The participants were allowed to use either overhand, mixed, or hook grip. Participants were instructed to pull upward on the barbell as hard and as fast as possible and to continue their maximal effort for 6 s. All participants were instructed to relax before the command “GO!” to avoid the precontraction. The force-time curve for each trial was recorded by a force plate (Kistler 9281CA, KISTLER, Winterthur, Switzerland) with a sample rate of 1,000 Hz (Comfort et al., 2015). Peak force was defined as the highest force achieved during the 6-s isometric test minus the participant’s body weight in Newtons. Additionally, force outputs at 30, 50, 90, 100, 150, 200, and 250 ms from the initiation of the pull were determined for each trial.

#### Vertical jump test

The vertical jump was used as a performance test to assess lower-limb power, including the height of CMJ and SJ (Nonnato et al., 2022). Series of SJs and CMJs were performed on a force platform three times (Kistler 9281CA, KISTLER, Winterthur, Switzerland). Participants were asked to jump three times and the higher height of a jump from each series was used for analysis. Take-off was strictly monitored by allowing no preliminary steps or movements. The force platform accurately recorded take-off and landing time and this allowed for the assessment of the duration of the flight phase and hence the calculation of CMJ and SJ height using the equation proposed by Bosco (Bosco et al., 1983).

#### PSAP and RSI test

PSAP and RSI were used to indirectly examine the ability of an athlete to use the stretch-shortening cycle (SSC) to improve their jump height and peak power during a vertical jump, which was often used as an indicator of lower-limb power performance (Suchomel et al., 2016). Indices from the jump data were PSAP and were calculated as follows:


$$\mathrm{PSAP}=\frac{\mathrm{CMJ}\mathrm{height}-\mathrm{SJ}\mathrm{height}}{\mathrm{SJ}\mathrm{height}}\times 100\%$$

RSI was measured by using a drop jump, performed starting from a standing position, with the hands placed on the hips. Participants stepped off the box with one foot, landing with two feet simultaneously on the force plate. As contact was made with the force plate participants immediately performed a vertical jump. The drop jumps (DJ) were carried out at heights of 45 cm. Participants were given three trials at each height with the best trial being used for analysis. RSI was calculated by dividing jump height (mm) by contact time (ms) (Barker et al., 2018).


$$\mathrm{RSI}=\frac{\mathrm{DJ}\mathrm{height}}{\mathrm{Ground}\;\mathrm{contact}\;\mathrm{time}}$$

### Statistical analyses

Experimental data were processed by IBM SPSS statistical software package (version 25.0, IBM, Chicago, IL, United States). Data were presented as means ± standard deviation (SD) and 95%CI. The level of significance was set at *p* < 0.05 for all tests. The normality of data distribution was confirmed by using the Shapiro–Wilk test, and those not normally distributed were confirmed by using the non-parametric Friedman test. To examine the effects of exercise training (i.e., RT and CT) on the performance of lower-limb strength and power, we performed one-way repeated-measure ANOVA. The dependent variables for each model were 1RM squat, IMTP, CMJ, SJ, PSAP, and RSI. The model factor was the time (pre- and post-intervention of week 1–8 and week 10–18). The absolute value of each test result was used to calculate the effect size (ES) for the within-group comparisons, represented as Cohen’s d. It was interpreted according to the following thresholds: <0.2 as trivial, 0.2–0.6 as small, 0.6–1.2 as moderate, 1.2–2.0 as large, and >2.0 as very large (Hopkins et al., 2009).

## Results

All participants completed all the training sessions and assessments, and their data were included in the analysis. All the data were normally distributed. Table 1 presents the demographic information of the participants (age: 23.55 ± 2.22 years; height: 169.44 ± 4.57 cm; weight: 60.59 ± 3.87 kg; training experience: 6.90 ± 2.08 years; BMI: 20.80 ± 0.99; body fat: 23.16 ± 2.32%).

**Figure 1:**
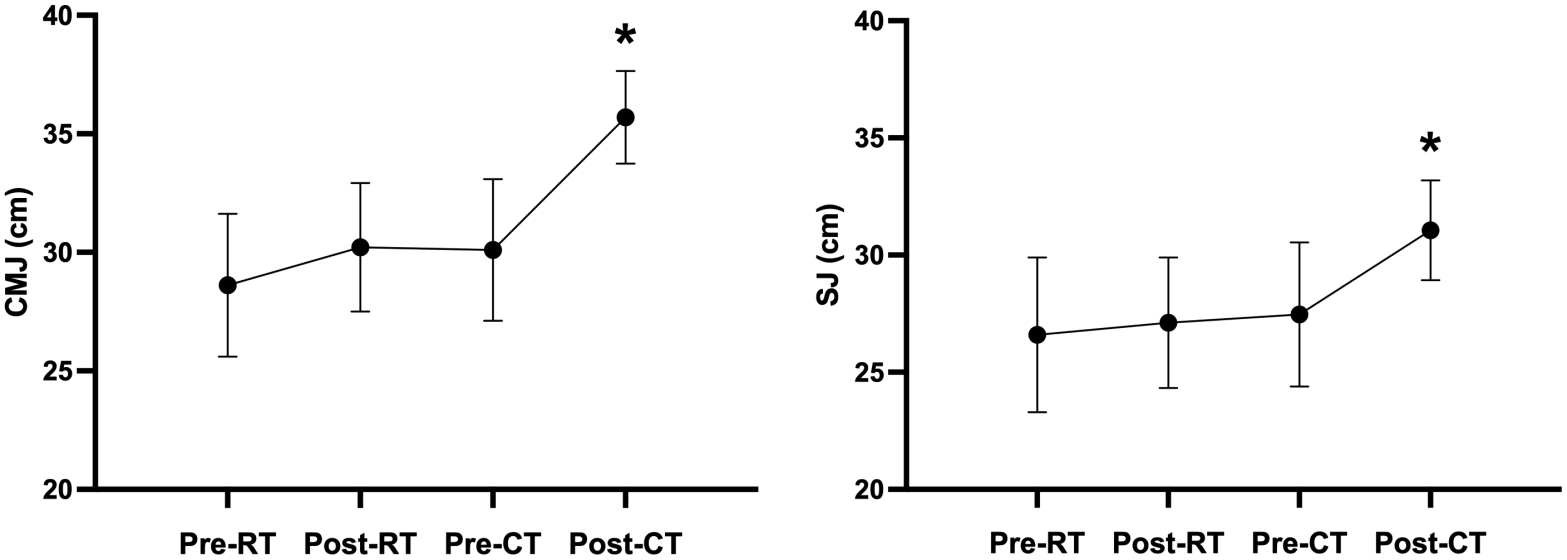
The assessment results of CMJ and SJ after CT intervention compared to all pre- and post-intervention. ^*^Statistically significant difference as compared to all the other pre- and post-interventions, *p* < 0.05.

The one-way repeated-measure ANOVA models showed in the Table 3 that: (1) The 1RM of squat was significantly improved (*p* < 0.001) after RT as compared to pre-RT. Such significant improvements were not observed in IMTP (*p* = 0.055), CMJ (*p* = 0.194), SJ (*p* = 0.692), PSAP (*p* = 0.087), and RSI (*p* = 0.238); and (2) after CT, 1RM of squat (*p* < 0.001), IMTP (*p* < 0.035), CMJ (*p* < 0.001), SJ (*p* < 0.008), and RSI (*p* < 0.006) were significantly improved as compared to pre-RT, post-RT, and pre-CT (Figure 1). Additionally, significant improvements in PSAP after CT were observed as compared to pre-RT (*p* = 0.003) and pre-CT (*p* = 0.027), but not to post-RT (*p* = 0.156).

## Discussion

This pilot study showed that CT following RT only can enhance lower-limb strength and power in elite female modern pentathlon athletes (as assessed by 1RM squat, IMTP, CMJ, SJ, and RSI), suggesting that this type of intervention would be an appropriate strategy to help maximize such functions in athletes of modern pentathlon, and ultimately help improve their performance, especially under the newly-established competition rules.

We observed that 1RM of squat was improved after completing both RT and CT, and after CT, the IMTP was significantly improved. Squat and IMTP mainly reflect lower-limb strength for quadriceps femoris and hamstring, which are linked to the performance of modern pentathlon athletes (Ko et al., 2021). The traditional RT focuses only on isometric contraction with slow velocity, and the conversion time between eccentric contraction and concentric contraction becomes longer. On the other hand, according to the principle of the force-velocity curve, the CT training protocol adopted larger-load (above 75% 1RM) exercises to enhance the strength-speed part of the curve, and used lower-load (below 50% 1RM) exercises to promote the maximum velocity part of the curve (Haff and Nimphius, 2012). CT intervention can simultaneously implement resistance exercises with larger-load and plyometric exercises with quicker velocity to improve multiple abilities of the entire force-velocity curve including strength-speed, peak power, speed-strength, and maximum velocity (Zatsiorsky et al., 2020; Weakley et al., 2021). It may thus induce significantly greater improvements specifically in 1RM of squat and IMTP by targeting quadriceps femoris and hamstring. These results suggested potential benefits from CT for lower-limb strength.

Significant improvements in CMJ, SJ, and RSI after completing CT, but not after RT, were observed. The CMJ and SJ reflect lower-limb power, associated with strength and speed performance (Jadczak et al., 2019). Previous studies showed that flight time and jump height mainly reflected lower-limb power, among which RSI was extremely important and an indicator of how efficiently athletes perform the SSC (Barker et al., 2018; Jeffreys et al., 2019). The results here suggest that CT can augment such efficiency of muscle action *via* plyometric exercises. Additionally, high-load RT in CT targets phosphorylation of the regulatory light chains, potentiated H-reflex response, and pennation angle of the muscle fibers to active more motor unit (Tillin and Bishop, 2009). However, no significant improvements in PSAP as induced by CT were observed. Suchomel et al. observed that PSAP may show vastly different results when comparing elite athlete ability and RSI will provide more information about how athletes use the SSC as compared with PSAP because of the incorporation of a timing component (e.g., ground contact time; Suchomel et al., 2016). Due to complicated factors of lower-limb power, more comprehensive assessments of power by characterizing multiple aspects of power are thus needed to future explore how the underlying physiological characteristics (e.g., electromyogram) affect power.

Several limitations of this pilot study should be noted. First, only 10 elite female participants were included, and the design of the study was not randomized with a control group. This is because we recruit athletes from the national team, and their daily training routine cannot be changed, we thus cannot implement randomization or control type of training for them. This limitation may thus raise potential issues of placebo or practice effects. Specifically, though many of the observed improvements in the performance can only be observed after completing CT, not RT, the contribution from RT still cannot be ignored, and such improvements might be related to the time effect (i.e., repeated training) instead of the unique benefits from CT. Future studies consisting of larger sample of participants (matched number of male and female) and using the design of randomization by including a control group are thus highly demanded to confirm the findings of our study. Second, studies with longer-term follow-up assessments using different CT protocols are needed to future determine the length that the benefits of CT can sustain, as well as the appropriate intensity and number of sessions of CT to maximize its benefits. Third, assessments that are linked to the performance of modern pentathlon athletes (e.g., the results of laser-run, swimming, and fencing) have not been used here; future studies are thus needed to determine the effects of CT on the match performance of modern pentathlon. Third, in this pilot study, though we asked participants to keep their ordinary daily nutritional intake without taking additional nutritional supplements or caffeine drinks, we did not record/track the daily nutritional intake participants, which may have potential impact on their performance. It is worthwhile to include this important information in the analysis of future studies Nevertheless, this pilot and non-randomized study provided preliminary evidence that CT might significantly augment lower-limb strength and power in elite modern pentathlon athletes.

## Data availability statement

The raw data supporting the conclusions of this article will be made available by the authors, without undue reservation.

## Ethics statement

The studies involving human participants were reviewed and approved by the study protocol was approved by the Beijing Sport University Institutional Research Commission (approval number: 2022132H). The patients/participants provided their written informed consent to participate in this study.

## Author contributions

ZQ, ZG, ML, LZ, and DB: research concept and study design. ZQ, ZG, LZ, and JZ: literature review, writing of the manuscript, and writing—original draft preparation. BL, ML, and DB: conceptualization and methodology. ML, LZ, and DB: formal analysis, investigation, and resources. ZQ, ZG, BL, and LZ: data collection, data analysis and interpretation, and statistical analyses. ZQ, ZG, LZ, DB, and JZ: writing—review and editing. All authors have read and agreed to the published version of the manuscript.

## Funding

This research was funded by the National Key Research and Development Project of China, under grants 2019YFF0301602-3 and 2019YFF0301803.

## Conflict of interest

The authors declare that the research was conducted in the absence of any commercial or financial relationships that could be construed as a potential conflict of interest.

## Publisher’s note

All claims expressed in this article are solely those of the authors and do not necessarily represent those of their affiliated organizations, or those of the publisher, the editors and the reviewers. Any product that may be evaluated in this article, or claim that may be made by its manufacturer, is not guaranteed or endorsed by the publisher.
